# Combined Prediction Model of Death Toll for Road Traffic Accidents Based on Independent and Dependent Variables

**DOI:** 10.1155/2014/103196

**Published:** 2014-12-31

**Authors:** Feng Zhong-xiang, Lu Shi-sheng, Zhang Wei-hua, Zhang Nan-nan

**Affiliations:** School of Transportation Engineering, Hefei University of Technology, Hefei, Anhui 230009, China

## Abstract

In order to build a combined model which can meet the variation rule of death toll data for road traffic accidents and can reflect the influence of multiple factors on traffic accidents and improve prediction accuracy for accidents, the Verhulst model was built based on the number of death tolls for road traffic accidents in China from 2002 to 2011; and car ownership, population, GDP, highway freight volume, highway passenger transportation volume, and highway mileage were chosen as the factors to build the death toll multivariate linear regression model. Then the two models were combined to be a combined prediction model which has weight coefficient. Shapley value method was applied to calculate the weight coefficient by assessing contributions. Finally, the combined model was used to recalculate the number of death tolls from 2002 to 2011, and the combined model was compared with the Verhulst and multivariate linear regression models. The results showed that the new model could not only characterize the death toll data characteristics but also quantify the degree of influence to the death toll by each influencing factor and had high accuracy as well as strong practicability.

## 1. Introduction

With the gradual progress of “Science and Technology Action Plan of Traffic Safety” and the implementation of “Law of the People's Republic of China on Road Traffic Safety,” the number of traffic accidents and the degree of injury have shown a decreasing trend since 2004; however, the death toll was still about 60 000 every year. Among the four traffic accident indicators, death toll has a direct effect on the sense of security and the degree of stability of the society, so understanding the death toll in the future will be of great guiding significance for making subsequent traffic management measures and policies and will play a guiding role in the development and orientation of traffic safety guarantee technology. Therefore, the agreement on the predictions of death toll has always been the key point of related studies [1–5]. However, traffic accident is difficult to forecast due to its randomness. And because the related forecasting methods are affected by various factors, the precision is difficult to guarantee.

The commonly used predicting methods include regression analysis method, exponential smoothing method, fuzzy analysis method, and time series method. In the area of traffic safety and accident, gray theory, Markov method, and artificial neural network are several major predicting methods. For example, on the basis of gray model GM (1, 1) for traffic accident prediction, Markov chain prediction method was introduced by Li et al. [6] and then the gray Markov prediction model was built by him. By making use of the advantages of artificial neural network such as strong nonlinear approximation, fuzzy reasoning, and self-learning, Dong and Shi [7] built the BP neural network prediction model of traffic accident. Zhang et al. [8] and others, using ARIMA model, did some research on the time series' stationarity of the mortality among 100 000 people in traffic accidents in China from 1970 to 1997 and used SPSS software to fit the model and made forecast. The conclusion was as follows: ARIMA model could improve prediction accuracy and could be applied to different kinds of nonseasonal and seasonal time series. Based on gray system theory and Markov theory, Zhao and Xu [9] and others used the system cloud gray model SCGM(1, 1) c to fit the general trend of the time series data of road traffic and put forward the gray weighting Markov model SCGM(1, 1) c which could be used to predict the number of traffic accidents. This model was suitable for dynamic prediction with short time series, less data, and not too big random fluctuations.

The above methods have their own characteristics, but each has its own defects; for example, using the GM (1, 1) model in gray theory, we can proceed from traffic accident data to analyze the characteristics and change law of the data and to predict the trend in the future. The model is easy to use and there is no need to consider other factors, but it can only describe monotonic changing process. If combining it with Markov theory, we can get a new model “gray Markov model,” which is applicable to random fluctuation process of traffic accidents; however, as for the model, there are no uniform standards for the classification of the system states. Artificial neural network is a kind of method which simulates the process of information input and decision-making output of human brains, during which process, the specific process of information processing and model building is not shown, which is very simple and convenient, but the accuracy is influenced by the data greatly. Multiple regression method can build a mathematical relationship between accident results and related factors and quantify the process and extent of the influence of various factors to accidents, whereas the accuracy of the model is comparatively poor, for the selection of factors is variable, and the future trends of the factors must be predicted prior to the final prediction of accidents, which means that the predicted data will be used as dependent variables of next prediction.

This paper concluded and analyzed both advantages and disadvantages of the above models. To begin with, it planned to use Verhulst model, most suitable to traffic accident in gray theory, to make a preliminary prediction on the basis of analyzing the characteristics of accident data [10, 11], which could characterize the changing trend of accidents; and meanwhile, in order to reflect the impact of other factors on traffic accidents, a multiple regression model of death toll caused by traffic accidents was built to analyze the dependent relationship of traffic accidents; then, for the purpose of combining the advantages of the two models, a combined prediction model of traffic death toll, based on independent and dependent variables, was built. This model could not only reflect the fluctuation law of the change of traffic accidents but also reflect the dependent law of traffic death toll caused by the interaction of multiple factors.

## 2. The Verhulst Model of Traffic Death Toll

### 2.1. Verhulst Model

In recent years, the development tendency of traffic death toll in China has shown a saturated S-shaped process, so it was suitable to use Verhulst model to make prediction [12]. The fundamental and process of Verhulst model building are as follows.

(*1) Model Building.* Let the original data sequence of traffic accident death toll be *X* ^(0)^ = (*x* ^(0)^(1), *x* ^(0)^(2),…, *x* ^(0)^(*n*)); *n* was the number of data.

According to the original data, one accumulated that generating operation data sequence *X* ^(1)^ of death toll was built as follows:

(1) $$\begin{array}{c} X^{\left(1\right)}=\left(x^{\left(1\right)}\left(1\right),x^{\left(1\right)}\left(2\right),\ldots ,x^{\left(1\right)}\left(n\right)\right). \end{array}$$

In the above formula, the relationship between *X* ^(1)^ and *X* ^(0)^ was *x* ^(1)^(*k*) = ∑_*i*=1_^*k*^ *x* ^(0)^(*i*), *k* = 1,2,…, *n*. *Z* ^(1)^ was the consecutive neighbor mean sequence of *X* ^(1)^, *Z* ^(1)^ = (*z* ^(1)^(2), *z* ^(1)^(3),…, *z* ^(1)^(*n*)):

(2) $$\begin{array}{c} z^{\left(1\right)}\left(k\right)=\frac{1}{2}\left(x^{\left(1\right)}\left(k\right)+x^{\left(1\right)}\left(k-1\right)\right). \end{array}$$

So

(3) $$\begin{array}{c} x^{\left(0\right)}\left(k\right)+\alpha z^{\left(1\right)}\left(k\right)=\mu {\left(z^{\left(1\right)}\left(k\right)\right)}^{2} \end{array}$$

was called gray Verhulst model. Consider that

(4) $$\begin{array}{c} \frac{\text{d}x^{\left(1\right)}}{\text{d}t}+ax^{\left(1\right)}=\mu {\left(x^{\left(1\right)}\right)}^{2} \end{array}$$

was the winterization equation of Verhulst model.

Let $\hat{\alpha }$ be the vector of parameter to be determined, and $\hat{\alpha }=\left(\begin{array}{c} a\\ \mu \end{array}\right)$, in which *a* was development gray number and *μ* was inner control gray number.

Discretizing formula (4) gives $Y=B\hat{\alpha }$.

Using the least square method to solve the problem gives $\hat{\alpha }={\left(B^{T}B\right)}^{-1}B^{T}Y$.

In the formula,

(5) $$\begin{array}{c} B=\left[\begin{array}{cc} -z^{\left(1\right)}\left(2\right) & {\left(z^{\left(1\right)}\left(2\right)\right)}^{2}\\ -z^{\left(1\right)}\left(3\right) & {\left(z^{\left(1\right)}\left(3\right)\right)}^{2}\\ \vdots & \vdots \\ -z^{\left(1\right)}\left(n\right) & {\left(z^{\left(1\right)}\left(n\right)\right)}^{2} \end{array}\right],\;\;Y=\left[\begin{array}{c} x^{\left(0\right)}\left(2\right)\\ x^{\left(0\right)}\left(3\right)\\ \vdots \\ x^{\left(0\right)}\left(n\right) \end{array}\right]. \end{array}$$

Then the parameter values of *a*, *μ* could be obtained. Substitute them into formula (3) to solve and get the solution of winterization equation. Namely, the time response function of accumulated generating sequence was

(6) $$\begin{array}{c} x^{\left(1\right)}\left(t\right)=\frac{\alpha x^{\left(1\right)}\left(0\right)}{\mu x^{\left(1\right)}\left(0\right)+\left(\alpha -\mu x^{\left(1\right)}\left(0\right)\right)e^{\alpha t}}. \end{array}$$

The time response formula of the model was

(7) x^(1)k+1=αx(1)(0)μx(1)(0)+(α−μx(1)(0))eαk,hhhhhhhhhhhhhhhhhhhhhhhhk=1,2…,n.

The inverse accumulated reduction formula was

(8) $$\begin{array}{c} {\hat{x}}^{\left(0\right)}\left(k+1\right)={\hat{x}}^{\left(1\right)}\left(k+1\right)-{\hat{x}}^{\left(1\right)}\left(k\right). \end{array}$$

(*2) Residual Test of the Model.* It is necessary to test the accuracy before building a prediction model, which can determine the validity. Residual test is a commonly used method for the test. In detail, model values and measured values are tested point by point.

Calculate the value of ${\hat{X}}^{\left(1\right)}\left(k\right)$ by the model and transfer ${\hat{X}}^{\left(1\right)}\left(k\right)$ to ${\hat{X}}^{\left(0\right)}\left(k\right)$ by inverse accumulated generation operation and then calculate the absolute and relative error sequences of the original sequence *X* ^(0)^(*k*) and ${\hat{X}}^{\left(0\right)}\left(k\right)$:

(9) $$\begin{array}{c} {\hat{X}}^{\left(0\right)}\left(k\right)=X^{\left(1\right)}\left(k\right)-X^{\left(1\right)}\left(k-1\right),\\ \Delta^{\left(0\right)}\left(k\right)=\left|X^{\left(0\right)}\left(k\right)-{\hat{X}}^{\left(0\right)}\left(k\right)\right|,\;k=1,2,\ldots ,n,\\ \varphi \left(k\right)=\frac{\Delta^{\left(0\right)}\left(k\right)}{X^{\left(0\right)}\left(k\right)}\times 100\%,\;k=1,2,\ldots ,n. \end{array}$$

### 2.2. Death Toll Forecast

Based on the data of traffic accident death tolls in China from 2002 to 2011 and the above calculation method of Verhulst model, a prediction model could be built. The specific data were shown in Table 1.

Make inverse accumulated operation of the original sequence of death toll

(10) X10=109381,104372,99217,98738,89455,81649,h73484,67159,65225,62387

and give the inverse accumulated operation sequence

(11) X1(1)=109381,−5009,−5155,−479,−9283,−7806,h−8165,−6325,−1934,−2838.

And its 1-iAGO neighbor generation sequence was

(12) Z(1)=106877,101795,98978,94097,85552,h77567,70322,66192,63806.

By calculation, *a* = 0.12238288; *μ* = 0.00000069 are given.

Substitute the above parameters into formula (7); the prediction model was as follows:

(13) x^(1)k+1=αx(1)(0)μx(1)(0)+(α−μx(1)(0))eαk=13386.362152680.07541568+0.04696720e0.12238288k.

According to the above formula, prediction of the death tolls from 2002 to 2011 was made once again, and the residuals were calculated. The details were shown in Table 2.

## 3. Multiple Linear Regression Method

### 3.1. Model Overview

The main idea of multiple linear regression method is to build correlation analysis of two or more dependent and independent variables. There were many related researches and the technology was very sophisticated. After the regression model was built, a statistic test of the model was necessary, including determination coefficient test (*R* ^2^ test), significance test of regression coefficient (*t*-test), and significance test of regression equation (*F* test). If the significant test of regression equation failed, it was possible that important factors were missed during the selection of independent variables, or the relationship between independent and dependent variables was nonlinear, in which situation the model should be rebuilt.

### 3.2. Analysis of Model Influence Factors

Road traffic system is mainly composed of human, vehicle, road, and environment, and each subsystem contains multiple factors. If one or more factors go wrong, the traffic safety will be discounted and the probability of traffic accidents will increase. Therefore, road traffic accident prediction needs to be analyzed from the above four systems in both macroscopic and microcosmic terms; the features of accidents should be considered, the factors related to accident greatly studied, and the process and inducements of accidents quantified. This paper studied the death toll law and the future development tendency of traffic accidents in China, which belonged to macroresearch, and thus this paper planned to select some macroindicators as factors, such as population, vehicle population, highway mileage, and passenger and freight turnover volume. The main reason for selecting macroindicators was that the above factors could reflect the overall degree of traffic activity. For example, with a large population base, the trip volume was relatively larger; the increase of vehicle population and highway mileage would encourage travelers to travel; passenger and freight turnover volume could directly reflect the frequent traffic behaviors of passengers and freights. Numerous traffic behaviors would increase the cardinal number of traffic accidents and had something to do with traffic accidents. Besides, influenced by policies and security technology as well as some other factors, the number of traffic accidents and death toll should be subject to change; however, these kinds of factors were difficult to quantify, and if quantification was unscientific, the correctness and precision of the prediction model would be affected, so this paper would not select relevant indicators for the time being.

### 3.3. Death Toll Forecast

With the traffic accident death tolls from 2002 to 2011 in China as dependent variables and the above relevant data as independent variables, a model was built. The detailed data were shown in Table 3.

SPSS 18.0 was used to build a multiple linear regression model, to calculate the correlation between the above factors and dependent variables—death tolls (see Table 4)—and to calculate determination coefficient *R* ^2^, test value *t*, and test value *f*. The details were shown in Table 4.

In Table 4, the minimum value of correlation coefficient between independent variables and death tolls was 0.890, which indicated that the above six factors had significant correlation with traffic death toll, so it was feasible to build a multiple linear regression model. In addition, the determination coefficients of the regression model were *R* ^2^ = 0.996, *F* = 67.431, which indicated that the regression degree of model equation to data was very high. The coefficient of each factor was shown in Table 5.

The equation of regression model could be obtained by the above coefficient values:

(14) Y=779909.386−9.153S1−5.403S2−0.147S3 +0.087S4−0.028S5+0.003S6.

In the equation, *Y* was death toll/person, *S*_1_ was vehicle population/10^4^ vehicles, *S*_2_ was population/10^4^ persons, *S*_3_ was GDP/10^8^ yuan, *S*_4_ was freight volume by road/10^4^ *t*, *S*_5_ was passenger volume by road/10^4^ persons, and *S*_6_ was road mileage/km.

The death tolls from 2002 to 2011 were predicted again by the above equation, and the prediction values as well as the relative errors could be seen in Table 6.

## 4. Combined Prediction Model of Traffic Death Toll

Suppose the combined prediction model is *f*(*x*) = ∑_*i*=1_^*m*^ *ω*_*i*_ · *f*_*i*_(*x*), in which *ω*_*i*_ is weighting coefficient of each model, *ω*_*i*_ ≥ 0, and ∑_*i*=1_^*m*^ *ω*_*i*_ = 1. The model in this paper was the combination of two models, so we could let *f*_1_(*x*) be the Verhulst model and let *f*_2_(*x*) be the multiple regression model to build a combined prediction model *f*(*x*) = *ω*_1_∗*f*_1_(*x*) + *ω*_2_∗*f*_2_(*x*).

In the above combined prediction model, weighting coefficient would affect the accuracy of the model directly, so the selection of reasonable weighting coefficient was very important. The selection methods included arithmetic average method, standard deviation method, mean square inverse method, analytic hierarchy process, and optimal weighting method. Arithmetic average was the simplest method, but with poorest reasonability, and could not reflect the differences between the models and the contributions to the final prediction results. As for analytic hierarchy process, the value of weighting coefficient must be assigned manually by relevant scholars, which was subject to subjective factors. The accuracy of optimal weighting method was very high, but the calculation was complicated; besides, the weighting coefficient might be negative, which had great limitations in practical application.

This paper determined weighting coefficient by Shapley method, a mathematical method which was proposed by Professor Shapley in 1953 and could be used to solve multiperson cooperation games by achieving a fair and efficient allocation of team total revenue between members [13]. The greatest advantage was that principles and results were easy to be deemed as fair by each partner and the result was easy to be accepted. The total error of combined prediction, generated for the reason of joint actions of each single prediction method in the process of combined prediction, could be regarded as a kind of “cooperation relationship” of the prediction methods for the same purpose.

### 4.1. Shapley Value Method

Suppose the combined prediction model contains *n* kinds of prediction methods, which could be denoted by *I* = {1,2,…, *n*}, *s* was any subset of *I*, *E*(*s*) was the combined error of this subset, the absolute value of error of the *i*th prediction method was *E*_*i*_, and the total error of the combined prediction was *E*. The values were as follows:

(15) $$\begin{array}{c} E_{i}=\frac{1}{m}\overset{n}{\underset{j=1}{\sum }}\left|e_{ij}\right|,\;\left(i=1,2,\ldots ,n\right),\\ E=\frac{1}{n}\overset{n}{\underset{i=1}{\sum }}E_{i}. \end{array}$$

In the above formulas, *m* was the number of samples and *e*_*ij*_ was the prediction error of combining *i*th prediction method with *j*th data.

The distribution formula of Shapley value was

(16) $$\begin{array}{c} E_{i}=\underset{si\in s}{\sum }\omega \left(\left|s\right|\right)*\left[E\left(s\right)-E\left(s-\left\{i\right\}\right)\right],\\ \omega \left(\left|s\right|\right)=\frac{\left(n-\left|s\right|\right)!\left(\left|s\right|-1\right)}{n!}. \end{array}$$

In the formula, *s* was the set containing prediction model *i*. |*s*| was the number of prediction models in the combination. *ω*(|*s*|) was weight factor which reflected the contributions of model *i* in the combined model. *s* − {*i*} was the removal model *i* in the combination modal.

The weight of each prediction method in the combination prediction was

(17) $$\begin{array}{c} \omega_{i}=\frac{1}{n-1}*\frac{E-E_{i}}{E},\;i=1,2,\ldots ,n. \end{array}$$

### 4.2. Weight Calculation of Prediction Model

According to the results of Tables 2 and 6, the total error of the combined prediction was *E* = 1/2∗(1.534 + 2.700) = 2.117.

Based on the concept of Shapley value, the “cooperation relationship” member involved in the total error apportion of the combined prediction model was *N* = {1,2}, and the combined errors of all of their subsets were *E*{1}, *E*{2}, *E*{1,2}, respectively. The values of the combined errors were average values of vector errors contained in the above subsets. See Table 7.

According to Shapley calculation method, the Shapley value of each member was obtained as follows:

(18) E1=0!1!2!E1−E1−1 +1!0!2!E1,2−E1,2−1=0.4755,E1=0!1!2!E2−E2−2 +1!0!2!E1,2−E1,2−2=1.6415.

The summation of the two members was *E*_1_ + *E*_2_ = 0.4755 + 1.6415 = 2.117 = *E*_3_, which indicated that the summation of two errors generated from two single prediction methods was equal to the total error *E*. This indicated that the calculation of the shared error of each method was correct. And the shared values indicated the precision of the prediction models. According to the above calculations and formula *ω*_*i*_ = 1/(*n* − 1)∗(*E* − *E*_*i*_)/*E*, the final weight of each single prediction method in the combined prediction model was

(19) $$\begin{array}{c} \omega_{1}=\frac{1}{2-1}*\frac{2.1170-0.4755}{2.1170}=0.7754,\\ \omega_{2}=\frac{1}{2-1}*\frac{2.1170-1.6415}{2.1170}=0.2246. \end{array}$$

Based on the above weights *ω*_1_ and *ω*_2_, the combined prediction model was got as follows:

(20) $$\begin{array}{c} f\left(x\right)=0.7754f_{1}\left(x\right)+0.2246f_{2}\left(x\right). \end{array}$$

### 4.3. Analysis of Model Accuracy and Result

#### 4.3.1. Prediction Value of Traffic Death Tolls in China from 2002 to 2011

With the above formulas of the combined prediction model to predict the traffic death tolls in China from 2002 to 2011, the results, shown in Table 8, could be obtained through calculations.

Among the above three models, Verhulst model could reflect the fluctuation of accident data, so there was a great change in its precision; however, the comprehensive error 2.700% was still very small. Multiple regression model was affected by multiple factors, and there were many original data for calculation, which could reflect the variation trends of practical data, so the accuracy of the model was very high. The maximum relative error was only 3.977% and the comprehensive error only 1.534%. The relative error of the combined prediction model lay between the errors of the above two models, and the comprehensive error was 2.205%. Though the accuracy was a little lower than that of multiple regression model, the model was applicable to medium and long term accident prediction because the results of this model could reflect the change rule of accident data, and the model adopted the thought of dependent relationship between the factors and the number of accidents in multiple regression model; therefore, it could not only reflect the tendency in the future qualitatively but also reflect the mathematical relationship between the factors and the model quantitatively.

#### 4.3.2. Prediction Value of Traffic Death Tolls in China from 2012 to 2013

The data from 2012 to 2013 are not used for modeling, so they are fit for verifying the accuracy of the model, and the related statistical data from 2012 to 2013 are shown in Table 9.

The traffic death tolls in China from 2012 to 2013 were calculated by formulas (13), (14), and (20), and the values and relative errors are shown in Table 10.

From Table 10, the death toll prediction value by multiple linear regression model in 2012 is 62402, and the relative error is 4.009%, but in 2013 they are 73962 and 32.035%. By analyzing the passenger volume by road in 2013, it fell to 52.107% compared with 2012. But from 2002 to 2011, passenger volume by road rose close to 10% a year. This may be a great relationship with the operation of China's high-speed rail in 2013, so there is a big gap between prediction result and reality in 2013. The results show that multiple linear regression model has erratic projection during political and economic instability. The stability of Verhulst model is higher than multiple linear regression model from Table 10, but it has a big relative error too, 13.112% and 14.471%, respectively, in 2012 and 2013. The death toll prediction value of combined model is very good, the relative errors are 9.267% in 2012 and 4.025% in 2013, and the results coincide with the truth, so the combined model has a high validity.

## 5. Conclusion

1. The occurrence of traffic accidents, with great randomness and burstiness, relates to human, vehicle, road environment, and other factors. It is difficult to predict the change rule of accidents with common models. Verhulst model is regarded as the prediction model closest to its own change rule; however, it cannot be used to describe the quantitative influence of other factors to itself.
2. By quantifying the mathematical relationships between multifactor variables and dependent variables, multiple regression model can reflect the objective law that traffic accidents are affected by lots of factors; however, the factors are difficult to choose, and predicted data are needed for prediction again, so the errors are usually very large.
3. Combining the above two methods, calculating weight coefficient of each model through Shapley value method, a combined prediction model can be built based on Verhulst model and multiple linear regression model. The combined model can not only describe the features of the data of accident fatalities but also quantify the impact of factors to death toll; in addition, the accuracy is very high and the model is very practical.

## Figures and Tables

**Table 1:** Traffic accident fatalities in China from 2002 to 2011.

Year	2002	2003	2004	2005	2006
Fatalities/person	109381	104372	99217	98738	89455
Year	2007	2008	2009	2010	2011
Fatalities/person	81649	73484	67159	65225	62387

**Table 2:** Predicted value by Verhulst model of traffic accident fatalities in China from 2002 to 2011.

Year	Fatalities/person	Predicted value/person	Relative error/%
2002	109381	109381	0
2003	104372	104176	0.188
2004	99217	98859	0.361
2005	98738	93468	5.337
2006	89455	88042	1.580
2007	81649	82621	1.190
2008	73484	77246	5.119
2009	67159	71955	7.141
2010	65225	66785	2.392
2011	62387	61769	0.991
Comprehensive error			2.700

**Table 3:** Related statistical data from 2002 to 2011.

Year	Fatalities/person	Vehicle population/10^4^ vehicles	Population/10^4^ persons	GDP/10^8^ yuan	Freight volume by road/10^4^ *t*	Passenger volume by road/10^4^ persons	Road mileage/km
2002	109381	2053.17	128453	120332.7	1116324	1475257	1765222
2003	104372	2382.93	129227	135822.8	1159957	1464335	1810000
2004	99217	2693.71	129988	159878.3	1244990	1624526	1871000
2005	98738	3159.66	130756	184937.4	1341778	1697381	3345200
2006	89455	3697.35	131448	216314.4	1466347	1860487	3457000
2007	81649	4358.36	132129	265810.3	1639432	2050680	3584000
2008	73484	5099.61	132802	314045.4	1916759	2682114	3730200
2009	67159	6280.61	133474	340902.8	2127834	2779081	3860823
2010	65225	7801.83	134091	401512.8	2448052	3052738	4008229
2011	62387	9356.32	134735	472881.6	2820100	3286220	4106387

**Table 4:** Correlation coefficient.

Independent variables	Correlation coefficient related to fatalities of traffic accidents
Vehicle population (10^4^ vehicles)	0.941
Population (10^4^ persons)	0.987
GDP (10^8^ yuan)	0.971
Freight volume by road (10^4^ *t*)	0.951
Passenger volume by road (10^4^ persons)	0.974
Road mileage (km)	0.890

**Table 5:** The coefficient value of each factor.

	Coefficient	Standard error	Value of *t*	Significance
Constant	779909.386	802935.957	0.971	0.403
Vehicle population (10^4^ vehicles)	−9.153	21.124	−0.433	0.694
Population (10^4^ persons)	−5.403	5.943	−0.909	0.430
GDP (10^8^ yuan)	−0.147	0.235	−0.625	0.576
Freight volume by road (10^4^ *t*)	0.087	0.141	0.618	0.580
Passenger volume by road (10^4^ persons)	−0.028	0.027	−1.054	0.369
Road mileage (km)	0.003	0.003	1.023	0.382

**Table 6:** The prediction value of multiple linear regression model of traffic death tolls from 2002 to 2011 in China.

Year	Fatalities/person	Prediction value/person	Relative error/%
2002	109381	110334	0.871
2003	104372	105103	0.700
2004	99217	97660	1.569
2005	98738	96681	2.083
2006	89455	89975	0.581
2007	81649	83033	1.695
2008	73484	72208	1.736
2009	67159	69830	3.977
2010	65225	64208	1.559
2011	62387	62035	0.564
Comprehensive error			1.534

**Table 7:** Errors of each subset.

Subset	*E*{1}	*E*{2}	*E*{1, 2}
Average value of errors	1.534	2.700	2.117

**Table 8:** The combined model prediction value of traffic death tolls in China from 2002 to 2011.

Year	Fatalities/person	Prediction value of combined model/person	Relative error/%	Relative error of Verhulst model/%	Relative error of multiple linear regression model/%
2002	109381	109595	0.196	0	0.871
2003	104372	104384	0.011	0.188	0.700
2004	99217	98590	0.632	0.361	1.569
2005	98738	94190	4.606	5.337	2.083
2006	89455	88476	1.094	1.580	0.581
2007	81649	82714	1.304	1.190	1.695
2008	73484	76114	3.579	5.119	1.736
2009	67159	71478	6.431	7.141	3.977
2010	65225	66206	1.504	2.392	1.559
2011	62387	61829	0.894	0.991	0.564
Comprehensive error			2.025	2.700	1.534

**Table 9:** Related statistical data from 2012 to 2013.

Year	Fatalities/person	Vehicle population/10^4^ vehicles	Population/10^4^ persons	GDP/10^8^ yuan	Freight volume by road/10^4^ *t*	Passenger volume by road/10^4^ persons	Road mileage/km
2012	59997	10933.09	135404	519470.10	3188475	3557010	4237500
2013	56017	12670.14	136072	568845.21	3076648	1853463	4356200

**Table 10:** The combined model prediction value of traffic death tolls in China from 2012 to 2013.

Year	Fatalities/person	Multiple linear regression model		Verhulst model		Combined model	
		Prediction value/person	Relative error/%	Prediction value/person	Relative error/%	Prediction value/person	Relative error/%
2012	59997	62402	4.009	52130	13.112	54437	9.267
2013	56017	73962	32.035	47911	14.471	53762	4.025
